# Prior contest experience exerts a long-term influence on subsequent winner and loser effects

**DOI:** 10.1186/1742-9994-8-28

**Published:** 2011-11-03

**Authors:** Yi-Ting Lan, Yuying Hsu

**Affiliations:** 1 Department of Life Science, National Taiwan Normal University, 88, Section 4, Ting-Chou Rd., Taipei 11677, Taiwan

**Keywords:** animal contest, winner-loser effect, information, fighting ability, *Kryptolebias marmoratus*

## Abstract

**Introduction:**

Animals are capable of using information from recent experiences to modify subsequent behavioral responses. Animals' ability or propensity to modify their behavior in the light of new information has repeatedly been shown to correlate with, or be influenced by, either their intrinsic competitive ability or their dominance experience - an influence which can be long-lasting. Using a mangrove killifish, *Kryptolebias marmoratus*, as the study organism, we investigated whether and if so how the effect of a winning or a losing experience one day prior to a dyadic contest was modulated by both competitive ability measured two months previously and a winning or losing experience forced on the contestants one month previously.

**Results:**

Winning/losing experience forced on the fish one month previously affected how they utilized information from their winning/losing experience one day before Test Day: Individuals that were randomly assigned a losing experience one month previously were more susceptible to the influence of their 1-day winning/losing experience than those assigned a winning experience. Competitive ability measured two months previously, winning/losing experience from one month previously and the winning/losing experience received one day previously all significantly influenced the fish's contest behaviors on Test Day, although only 2-month competitive ability significantly influenced escalation duration, indicating that it was still a good index for the fish's competitive ability two months later.

**Conclusions:**

These results suggest that the value to the fish of information from a recent win or loss depends on the outcome of their past contests and show that contest experience has a long-term effect on contest behavior.

## Introduction

There is a rich literature demonstrating that animals can incorporate information from prior experiences into future behavioral decisions. In three-spined sticklebacks (*Gasterosteus aculeatus*) [1] and zebra finches (*Taeniopygia guttata*) [2], for instance, females' attraction to males was influenced by their previous experience with other males: females became more choosy after being exposed to better-quality males. Interestingly, male zebra finches adjust their courtship intensity based on the attention they receive from the females, even if the circumstances are manipulated so that this attention does not depend on the males' quality [3]. Moreover, patch choice in least chipmunks (*Tamias minimus*) and golden-mantled ground squirrels (*Spermophilus lateralis*) depended on the combined effect of their multiple previous experiences in the patches [4].

An individual's sensitivity to new information and readiness or capability to modify behavior after being exposed to new information can be closely associated with its intrinsic competitive ability and its dominance experience. In starlings (*Sturnus vulgaris*), for instance, individuals with better intrinsic competitive ability performed better in foraging-related learning tasks [5]. Similarly, dominant chickadees (*Poecile gambeli*) showed better spatial memory than subordinates in tasks relating to recovery of hidden food [6], and dominant male meadow voles (*Microtus pennsylvanicus*) had better spatial-learning ability than subordinates in water-maze tests [7]. Interestingly, the better spatial-learning ability of dominant mice (*Mus musculus*) was detected only after, not before, dominance status was established [8], indicating that it was probably the difference in contest experience, rather than the difference in intrinsic competitive ability between the dominants and subordinates that caused the differences in their performance in the food-tracking maze tests.

Although dominant individuals appear to perform better in spatial learning and food-reward associative learning (also see [9,10]; but see [11]), subordinates might be more sensitive to information associated with social learning and predation risks. In household dogs (*Canis familiaris*) [12], for instance, subordinates outperformed dominants in obtaining an object behind a fence after observing a dog demonstrator performing the task but there was no difference in the subordinates' and the dominants' performance after observing a human demonstrator, indicating that the source of the information could play an important role in animal learning. In addition, subordinate crabs (*Chasmagnathus granulatus*) showed higher memory retention than dominants in tests involving visual danger stimuli [13]. Because the difference between the dominant and the subordinate crabs occurred only after, not before, the establishment of the dominance relationship, it was probably the experience of winning/losing and not any intrinsic properties that stimulated the losers' memory. These differences in learning performance between dominants and subordinates could be long lasting, although most of the studies quoted above did not examine this. The impairment of spatial-learning ability in subordinate mice (outbred albino strain CD-1) subjected to high levels of aggression, for instance, persisted even after they were housed alone for more than 13 weeks [14]. It seems likely that competitive ability and dominance status could influence animals' propensity to incorporate information acquired in the past into future contest decisions given that this has been shown to apply to so many other contexts (mating, foraging, spatial learning etc.), but this has not yet been tested.

Like other behavioral decisions, an individual's choices in contests are influenced by experience [15-18]: after a recent win, an individual tends to behave more aggressively and enjoys an elevated chance of winning again (winner effect); after a recent loss, however, an individual tends to become more submissive and suffers a higher chance of losing again (loser effect). Winner and loser effects are often considered to result from individuals using information from previous wins or losses to raise or lower their assessments of their own fighting ability [19,20], which in turn influences their subsequent contest decisions. As discussed above there have been no previous studies of the possible influence of either competitive ability or dominance experience on how individuals incorporate information from a recent contest into their subsequent contest decisions.

Dominance experience or status is often loosely defined in previous studies; some use 'dominants' and 'subordinates' to refer to individuals cohabiting for some extended periods which have probably established stable aggressor-defender relationships [8,12,14], while others use 'dominants' and 'subordinates' simply to refer to winners and losers of contests, respectively, even where winners and losers are separated shortly after the contests are resolved [7,13]. In the field, dominant (subordinate) individuals probably have better (worse) intrinsic competitive ability and, at the same time, tend to win (lose) fights. In this study, we therefore aimed to explore whether and if so how the effect of a recent win or loss on contest behavior might differ between individuals with different competitive ability and, separately, with different contest experience. We used a hermaphroditic mangrove killifish (*Kryptolebias marmoratus*) as the study organism.

Individuals of *K. marmoratus *display both winner and loser effects in contests [17,21,22]. The effects of a winning or a losing experience on contest behavior and outcome appear to become undetectable within four days when examined by staging contests between individuals with a recent winning or losing experience and naïve opponents [23]. However, when both individuals of a contest pair won their last contest more than a month previously, they were more likely to escalate contests into physical fights and persisted longer in contests than when they both lost their last contest [17,24,25]. These trends were significant even if the fish were given another contest experience prior to the staged contests [17]. These results indicate that competitive ability and/or the contest experience acquired one month previously could play an important role in the fish's current contest decisions and that these influences can survive the influence of intervening contest experiences. They do not, however, allow us to tell whether the effect arises from intrinsic competitive ability or from the influence of prior contest experience. Building on these findings, this study examined the impact of an individual's intrinsic competitive ability and, separately, its contest experience from a month previously on whether and if so how the fish used the information from a recent contest to modify its behavioral responses in subsequent contests. In other words, we examined whether winner and loser effects varied according to the animals' intrinsic competitive ability and, separately, whether they varied according to the animals' experience of winning or losing another contest one month previously.

**Figure 1:**
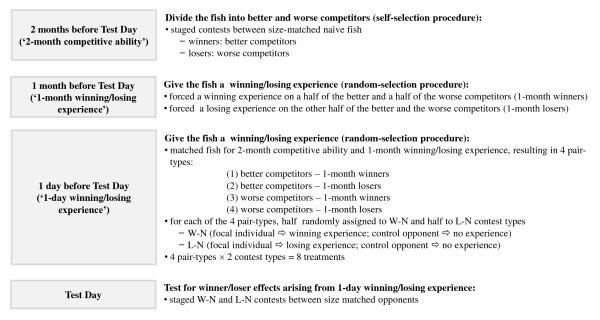
**Experimental procedures**.

To achieve these goals, two months prior to dyadic contests (Test Day), size-matched individuals from the same strain of *K. marmoratus *with no prior fighting experience were allowed to fight between themselves. The outcomes of these self-selection procedures [26] were used to divide them into better (winners) and worse (losers) competitors (Figure 1). This procedure is referred to hereafter as the '2-month competitive ability' procedure. The better and worse competitors from the 2-month competitive ability procedure differed in both intrinsic competitive ability and contest experience [26]. Differences in the behavior of these two groups of fish could therefore be caused by either or both factors, a fact which was taken into consideration in data interpretation. One month prior to Test Day, half of the better competitors were randomly chosen to receive a winning experience (1-month winners) by fighting with a smaller, habitual loser and the other half to receive a losing experience (1-month losers) by fighting with a larger, habitual winner using random-selection procedures [26]. The same process was applied to the worse competitors. This process (carried out one month prior to Test Day) is hereafter referred to as the '1-month winning/losing experience'. One day prior to Test Day, pairs of fish matched for their size, strain, 2-month competitive ability and 1-month winning/losing experience were randomly assigned to one of two contest types: W-N (in which the focal individual received a winning experience and the control opponent received no contest experience) or L-N (in which the focal individual received a losing experience and the control opponent received no contest experience). On the same day (one day before Test Day) one individual in each pair was chosen at random to be the focal individual. It then received the appropriate winning or losing experience (hereafter referred to as '1-day winning/losing experience'). The following day (Test Day), contests were staged between the two individuals of the pair. Contest behaviors of individuals of the W-N and L-N contest types were used to examine whether the winner and loser effects arising from the 1-day winning/losing experience were influenced by the fishes' 2-month competitive ability and their 1-month winning/losing experience. The experiment therefore had a total of eight treatment groups (2 '2-month competitive-ability' types × 2 '1-month experience' types × 2 '1-day contest' types). These treatment groups were replicated for all five strains of the fish in our laboratory (which were originally collected from different geographical areas) so that the findings would be relevant to the species as a whole and not restricted to a single strain. Since the studies of household dogs and crabs presented earlier found subordinates to be more inclined to learn from conspecifics [12] and more sensitive to risk-related information [13], we predicted that individuals with worse 2-month competitive ability and/or with 1-month losing experience would be more sensitive to the 1-day winning/losing experience and thus display stronger winner and loser effects than individuals with better 2-month competitive ability and/or with 1-month winning experience.

## Results

### The winner/loser effects arising from 1-day winning/losing experiences were modulated by 1-month winning/losing experience but not by 2-month competitive ability

A total of 480 contests were staged (8 treatments × 60 contests per treatment).

**Figure 2:**
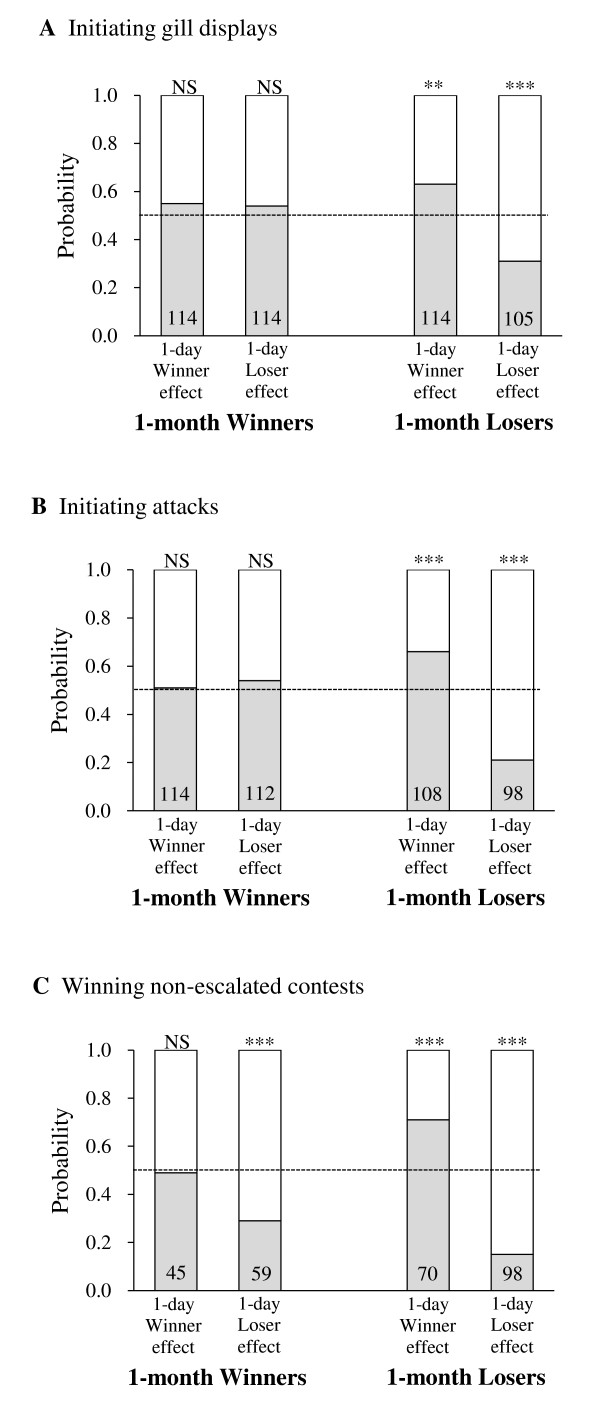
**Influence of 1-month winning/losing experience on the importance of 1-day winning/losing experience**. The importance of the 1-day winning (winner effect) or losing (loser effect) experience on the probability of (A) initiating gill displays, (B) initiating attacks and (C) winning non-escalated contests when both individuals of a contest pair were subjected to either winning or losing experience treatment one month prior to Test Day. The shaded portion of the bars represents the probability that the focal individual would carry out the relevant behavior (initiate displays, initiate attacks or win non-escalated contests) and the clear portion shows the probability for its control opponent. (The focal individual is the contestant that received a winning or a losing experience one day prior to Test Day; its control opponent is the animal that received no experience one day prior to Test Day.) NS *P *> 0.05; * *P *≤ 0.05; ** *P *≤ 0.01; *** *P *≤ 0.001; binomial test for whether the behavior concerned showed a significant winner or loser effect for the 1-month dominant or subordinate experience. The sample size for each bar is presented on the bottom of the bar. (Sample sizes differ because not all contests involved all behaviors).

Multiple logistic regression (Table 1) showed that when fighting with their control opponents, the focal individuals' contest strategies were significantly influenced by the 1-day winning/losing experience: focal individuals that received a 1-day losing experience were less likely to initiate gill displays (P < 0.001) or attacks (P < 0.001) or to win non-escalated contests (P < 0.001) than those that received a 1-day winning experience. These effects were, however, modulated by the outcome of the contests that focal individuals experienced one month previously, as indicated by the significant interaction between1-month winning/losing experience and 1-day winning/losing experience (initiating gill displays: P = 0.001, initiating attacks: P < 0.001, winning non-escalated contests: P = 0.003). The 1-day winning/losing experience had more influence on contest behavior in contest pairs comprising 1-month losers than in those comprising 1-month winners (Figure 2A-C). In 1-month losers, both winner and loser effects were detected (binomial tests) for initiating gill displays (winner effect: P = 0.003; loser effect: P < 0.001), initiating attacks (winner effect: P < 0.001; loser effect: P < 0.001) and winning non-escalated contests (winner effect: P < 0.001; loser effect: P < 0.001). In 1-month winners, however, only a loser effect was detected and then only for winning non-escalated contests (P < 0.001).

**Table 1:** Influence of 2-month competitive ability and 1-month winning/losing experience on 1-day winner/loser effect

		Initiating gill displays (*n *= 447)			Initiating attacks (*n *= 432)			Winning non-escalated (*n *= 272)		
		
Variable	*df*	b±SE	*χ^2^*	* P*	b±SE	*χ^2^*	* P*	b±SE	*χ^2^*	* P*
2 month-CA(worse)	1	-0.10±0.20	0.23	0.631	-0.02±0.21	0.01	0.905	0.23±0.31	0.55	0.459
1 month-W/L(losing)	1	-0.29±0.20	2.16	0.142	-0.38±0.21	3.47	0.063	0.01±0.29	0.00	0.972
1 day-W/L(losing)	1	-0.70±0.20	12.91	<0.001*	-0.87±0.21	18.51	<0.001*	-1.98±0.29	51.84	<0.001*
2 month-CA × 1 d-W/L	1	0.11±0.39	0.08	0.772	-0.03±0.41	0.01	0.935	0.88±0.61	2.10	0.147
1 month-W/L × 1 d-W/L	1	-1.25±0.39	10.15	0.001*	-2.06±0.42	25.69	<0.001*	-1.74±0.58	9.15	0.003*
Size	1	0.06±0.04	1.82	0.177	0.01±0.04	0.02	0.902	-0.01±0.07	0.05	0.826
Strain	4		6.50	0.165		0.31	0.989		3.65	0.456

Multiple logistic regression (likelihood ratio *χ^2 ^*statistic) modeling the influence of competitive ability measured two months previously (2 month-CA; the pairs comprising better competitors were the baseline group), contest experience received one month previously (1 month-W/L; the pairs where both contestants received a winning experience were the baseline group) and contest experience received one day previously (1 day-W/L; the W-N pairs where the focal individuals received a winning experience were the baseline group) on the contest behaviors of the focal individuals when fighting against the control opponents, controlling for the body size and the strain type of the contest pairs.

2-month competitive ability had no influence on the importance of the 1-day winning/losing experience as none of the interaction effects was significant (P ≥ 0.147, Table 1).

### 2-month competitive ability, 1-month winning/losing experience and 1-day winning/losing experience all significantly influenced contest behavior on Test Day

Although 2-month competitive ability did not influence the magnitude of the winner or loser effect arising from the 1-day winning/losing experience, it did significantly affect the fish's fighting behaviors on Test Day (Table 2): contest pairs comprising individuals with worse competitive abilities took longer to display (P = 0.003) and attack (P < 0.001) and escalated for shorter time periods (P = 0.035) than contest pairs comprising individuals with better competitive abilities.

**Table 2:** Effect of 2-month competitive ability, 1-month winning/losing and 1-day winning/losing experience on contest behaviors

		Latency to gill displays (*n *= 447, Ddf = 436)			Latency to attacks (*n *= 432; Ddf = 421)			Escalation duration (*n *= 208; Ddf = 197)		
		
Variable	*Ndf*	b±SE	*F*	* P *	b±SE	*F*	* P*	b±SE	*F*	* P*
2 month-CA(worse)	1	0.40±0.14	8.62	0.003*	0.54±0.11	23.30	<0.001*	-0.41±0.19	4.51	0.035*
1 month-W/L(losing)	1	0.32±0.13	5.90	0.016*	0.47±0.11	18.37	<0.001*	-0.02±0.19	0.01	0.906
1 day-W/L(losing)	1	0.40±0.13	8.83	0.003*	0.58±0.11	27.24	<0.001*	-0.30±0.19	2.37	0.125
2 month-CA × 1 d-W/L	1	-0.30±0.27	1.28	0.258	-0.00±0.22	0.00	0.992	-0.24±0.39	0.36	0.547
1 month-W/L × 1 d-W/L	1	0.05±0.27	0.04	0.843	0.27±0.22	1.46	0.228	0.66±0.40	2.69	0.103
Size	1	0.09±0.03	9.80	0.002*	0.05±0.02	4.13	0.043*	-0.02±0.04	0.17	0.679
Strain	4		6.88	<0.001*		1.14	0.338		5.20	<0.001*

Multiple linear regression modeling the influence of competitive ability measured two months previously (2 month-CA; the pairs comprising better competitors were the baseline group), contest experience received one month previously (1 month-W/L; the pairs where both contestants received a winning experience were the baseline group) and contest experience received one day previously (1-day-W/L; the W-N pairs where the focal individuals received a winning experience were the baseline group) on the latency to gill displays, latency to attacks and escalation duration, controlling for the body size and the strain type of the contest pairs. Ndf: numerator degree of freedom; Ddf: denominator degree of freedom.

1-month winning/losing experience also significantly affected latency to gill displays (P = 0.016) and attacks (P < 0.001) such that 1-month loser pairs took longer to initiate displays and attacks than 1-month winner pairs. The 1-month winning/losing experience, however, did not significantly influence escalation duration (P = 0.906). Similarly, 1-day winning/losing experience significantly affected latency to gill displays (P = 0.003) and attacks (P < 0.001) such that L-N pairs took longer to initiate displays and attacks than W-N pairs, but also did not significantly influence escalation duration (P = 0.125).

**Figure 3:**
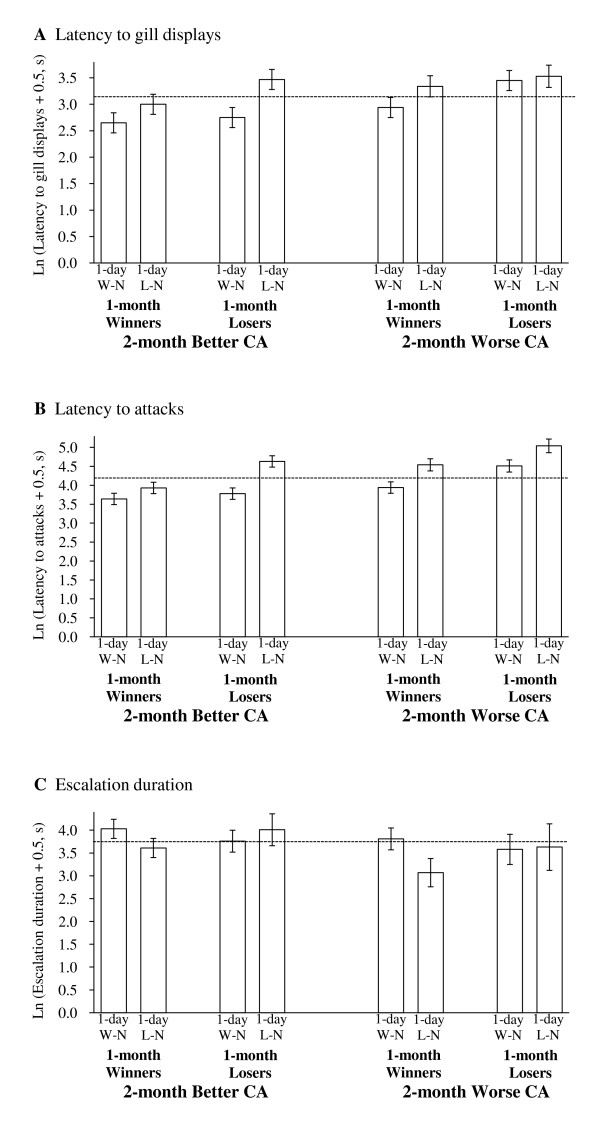
**Contest behavior for the eight treatment groups**. The bars represent the mean responses (ln transformed, mean ± SE) for (A) latency to gill displays, (B) latency to attacks and (C) escalation duration for the eight treatment groups. The dashed line indicates the grand mean.

The mean treatment responses (natural log transformed) for the contest behaviors measured are summarized in Figure 3 for the eight treatment groups. Although 2-month competitive ability, 1-month winning/losing experience and 1-day winning/losing experience all had important influences on some or all of the contest behaviors measured on Test Day, none of the interaction terms (P ≥ 0.103) was significant (Table 2).

Although not the primary interest of this study, the fishes' contest behaviors varied according to their body size and strain type: the larger the pairs, the longer the time before one of them initiated gill displays (P = 0.002) or attacks (P = 0.043). Individuals of the VOL strain were the most aggressive, being fastest to initiate gill displays and persisting longest in escalation; the other four strains did not differ significantly from each other in these behavioral measures (Tukey pair-wise comparisons).

## Discussion

This study discovered 1) that whether information from a recent win or loss is incorporated into subsequent contest decisions is influenced by the outcome of fights that the contestants experienced one-month previously but not by the contestants' intrinsic competitive ability and 2) that the influence of winning and losing experiences on contest behavior persists considerably longer than previously reported (more than one month).

The behavior of individuals randomly assigned to receive a losing experience one month previously was more susceptible to the influence of a one-day old win or loss than the behavior of those that received a winning experience. Interestingly, intrinsic competitive ability measured two months previously did not have much effect on how the fish responded to the 1-day wining/losing experience, even though the process of separating the better from the worse competitors two months prior to Test Day also led them to experience a victory or a loss. The influence of 1-month winning/losing experience on 1-day winning/losing effects, and the lack of influence from the 2-month winning/losing experience, could be explained either because of time decay or sequence. i.e., the winning/losing experience from the 2-month competitive ability process may not have influenced the 1-day winning/losing effect, either because it was too old, or because its influence was supplanted by the more recent 1-month winning/losing experience.

The influence of contest experience and the lack of influence from intrinsic competitive ability on how the mangrove killifish fish utilized information from the 1-day winning/losing experiences in our study suggest that the fish's propensity to modify contest strategy after a win/loss is a context dependent trait rather than a specific trait, present in some species but not in others. Prior winning and losing experiences are thought to influence how an individual evaluates its fighting ability relative to that of others in the population [15,19]. It therefore appears that it is not an individual's actual fighting ability but is its evaluation of its relative fighting ability that influences how *K. marmoratus *utilizes the newly acquired information on the same subject. Being modulated by dominance experience rather than intrinsic ability allows individuals to adjust their behavior according to how their ability relates to that of others in the local population. This could provide flexibility to individuals that live in populations with unpredictable distributions of fighting ability, where intrinsic ability would not serve as a reliable indicator of an individual's rank in the population.

That the importance of a recent winning or losing experience could differ systematically between groups of conspecifics is rarely explored empirically, despite the fact that winner and/or loser effects have been reported for animals of a wide range of taxa [15,16]. In California mice (*Peromyscus californicus*), the only example known to us, the usefulness of information from winning experiences was concluded to depend on the contest environment: individuals displayed a winner effect only if the winning experiences occurred in their home cages and not if they occurred in unfamiliar cages [27]. This supports the conclusion from our results that the degree to which an individual's behavior is influenced by winning and/or losing experiences appears to be phenotypically plastic - influenced by extrinsic factors including the environment at the time of the wins or losses or by the animal's previous experience. Although no empirical evidence has been presented, it is not unlikely that intrinsic factors such as contestants' age or development stage also play important roles in deciding whether and if so how individuals utilize the information from a recent win or loss [15,18]. More extensive exploration of factors important to the effects of recent winning/losing experiences should facilitate a better overall understanding of the adaptive value of the information from these experiences.

Dominant individuals often perform better than subordinates in spatial-learning and food-reward associative learning tests [5-8]. Subordinates, however, appear to be more receptive to information obtained from observing [12] or interacting with (our study) conspecifics, or to show better memory in visual-danger associative-learning tests [13]. Most studies, however, do not separate the effect of intrinsic competitive ability from the effect of winning/losing experience. i.e., in most studies the dominant individuals under investigation have a better than average competitive ability and a winning experience while subordinate individuals have a worse competitive ability and a losing experience. From the results of studies that did distinguish between the two factors, it appears that both competitive ability and contest experience may influence an individual's propensity to learn, although not necessarily in the same animal. Mice's (*Mus musculus*) ability to learn a maze task [8] and crabs' (*Chasmagnathus granulatus*) memory ability [13], for instance, are shown to be influenced by contest experience, while male meadow voles' (*Microtus pennsylvanicus*) spatial learning ability is related to their competitive ability [7].

Perhaps dominance status influences an individual's investment in different types of activities: dominant individuals invest more in resource acquisition and pay more attention to their physical environment whereas subordinate individuals invest more in danger avoidance and pay more attention to the activity of, and their interaction with, predators and conspecfics. This difference in investment between dominants and subordinates could be adaptive. For instance, subordinate individuals in the natural environment tend to have worse competitive ability and be weaker than the population average and are subjected to high risk of being attacked by conspecifics or targeted by predators. It therefore pays for them to monitor other individuals in the vicinity closely, to learn about their aggressiveness and gain information which may be useful in deciding whether or how to interact with them. Dominant individuals, on the other hand, are probably stronger than the population average and do not need to worry as much about being picked on by others. They can therefore afford to allocate more of their attention to exploring the physical environment and finding more resources. The results of our study, that the behavior of the fish randomly assigned to receive a losing experience one month previously was more susceptible to the influence of a one-day old win or loss than the behavior of those that receive a winning experience, could be a consequence of losing experience shifting the fish's attention more toward social interactions. More comparative studies on the performance of dominant and subordinate individuals when exposed to different types of information will allow us to have a better picture of the possible causes and functions of the differences in their performances.

Dominant and subordinate individuals are usually characterized by the difference in their aggressiveness, such that dominants consistently behave more aggressively than subordinates across different contexts (e.g., feeding, mating). Aggressive individuals are often found also to be bolder (e.g., more likely to engage in predator inspections) and more proactive (e.g., having a higher tendency to explore an unfamiliar environment) while less aggressive individuals tend to be more shy and reactive (i.e., paying careful attention to external stimuli and adjusting cautiously to changes in the environment). These correlations are known as behavioral syndromes [28]. These conclusions on behavioral syndromes (that aggressive animals are bolder and more proactive) appear consistent with the general finding that dominant individuals often perform better in spatial-learning and food-reward associative learning while subordinate individuals are more responsive to information derived from social interaction. The results of our study, that past winning and losing experience has a long-term effect on both an individual's aggressiveness and its responsiveness to new experiences, raise the question of whether aggressiveness and its relationships with boldness and exploratory behavior are stable traits (i.e., animal personality: [29,30]). To consider this question, we should bear in mind that the types of contest experience (win or loss) an individual receives in the natural environment are probably highly dependent on its competitive ability. For instance, a stronger individual probably tends to win fights and consequently becomes more aggressive and has access to more resources, further increasing its chance of winning future contests. Through this positive feedback, an individual's aggressiveness relative to that of others in the population and the relationships between aggressiveness and boldness and exploratory behavior could remain relatively stable over time (see [31] for more discussion). Experience effects, in this case, enhance the stability of an individual's personality (in terms of aggressiveness) rather than disrupting it.

Our study also revealed that competitive ability measured two months previously, winning/losing experience acquired one month previously and winning/losing experience acquired one day previously all significantly influenced how fast the fish displayed and attacked on Test Day. Only competitive ability, however, significantly influenced escalation duration: better pairs of competitors persisted longer in escalation than worse competitors. Escalation involves mutual physical interaction between the two contestants. Prolonged escalation duration is energetically demanding [32] and costly and is therefore a good indicator of the contestants' competitive ability on Test Day. This result shows that competitive ability measured two months previously still serves as a reasonable index for the fish's competitive ability two months later. The result that 1-month and 1-day contest experience did not significantly affect this behavior is consistent with the hypothesis that contest experience influences an individual's evaluation of its fighting ability but not its actual fighting ability [15,19].

It is rather surprising that the contest experience acquired in a random-selection procedure one month previously (i.e., winning/losing experience unrelated to underlying fighting ability) still had a significant effect on contest behaviors. Previous studies that employed random-selection procedures to test the longevity of winner/loser effects usually concluded them to be rather short-lived (from less than an hour to a few days) [33-36], excluding studies that trained the study animals multiple times or for prolonged periods. For instance, the effect of a winning experience lasted for less than three hours in sticklebacks (*Gasterosteus aculeatus*) [36] and less than an hour in pumpkinseed sunfish (*Lepomis gibbosus*) [33]. The effect of a losing experience usually lasts longer, but the effect started to decay in six hours in sticklebacks [36] and became undetectable in seven days in the copperhead snakes (*Agkistrodon contortrix*) [35]. A recent study on the contest behavior of *K. marmoratus *showed that the effect of winning or losing experience disappeared within four days [23], which may appear to be inconsistent with the finding of this study. Differences in methodology between the present and previous studies might partly explain the differences in findings. In previous studies, study animals with different contest histories were given a winning (W individual), a losing (L individual) or no recent contest (naïve opponent) experience. Contests were then staged between the W individuals and naïve opponents and between the L individuals and naïve opponents to test the significance of winner and loser effects after different periods of time. This was also the case in the studies of sticklebacks, pumpkinseed sunfish and copperheads mentioned earlier. In our current study, the two individuals of a contest pair had the same 1-month winning/losing experience (i.e., either both were winners or both were losers). We then compared the interactions between two 1-month winners with those between two 1-month losers (Table 2, 1 month-W/L(losing)). Contrasting the interactions between two 1-month winners with the interactions between two 1-month losers should allow us better to detect any influence of previous contest experience on the fish's contest behavior than comparing the behavior of a winner or a loser with a naïve opponent. Furthermore, this study used naïve fish that had never fought previously. Each of the study individuals received exactly three contest experiences prior to Test Day and the results of all three contests were included in the regression models. This experimental procedure minimized the influence of unknown contest histories on the fish's contest behavior, to make the influence of each of the three contest experiences more easily detectable. Overall, the difference in the conclusions between current and previous studies about the longevity of experience effects in *K. marmoratus *may indicate that the detectability of the effect of a winning/losing experience depends on the experimental procedures and the life history of the study individuals, which should be taken into consideration in future studies on these effects.

## Conclusions

Using a mangrove killifish, our study showed that the outcome of a contest one month previously can determine the extent to which a 1-day winning/losing experience influences current contest decisions. The 1-month losers were more susceptible than the 1-month winners to the influence of the 1-day winning/losing experience. Our study also revealed that competitive ability measured two months previously was still a good indicator for competitive ability two months later, indicating that it is a relatively stable trait in the fish. Moreover, the study showed that the effect of a winning and/or losing experience can last for more than a month in the fish. The difference in the adaptive value of information from recent contest experiences to previous winners and losers and the physiological mechanisms mediating these differential effects in the fish are yet to be investigated.

## Materials and methods

### Study species and animal care

*Kryptolebias marmoratus *is an internally self-fertilizing hermaphroditic fish [37] living in mangrove areas, distributed from Belize Central America to Florida [38]. Natural populations mainly consist of isogenic homozygous hermaphrodites with very low incidence (< 1%) of males, although an out-crossing heterozygous population with approximately 20% males has been discovered in Twin Cays, Belize [39]. The fish is often found in crab burrows and small ephemeral pools [40,41]. It has an epidermal capillary bed [42] which enables it to respire through air, be semiterrestrial and travel between locations by flipping or slithering through wet pebbles and mud [40,43]. The fish is aggressive in both the field and laboratory [43,44]. Two individuals confined in a small aquarium (12 × 8 × 20 cm^3^) usually establish a dominant-subordinate relationship in an hour [25] and, if a shelter is provided, the dominant individual of a pair is always the one that enters and defends the shelter [45].

This study used five strains of *K. marmoratus *from various geographical areas (DAN2K: Dangria, Belize; HON9: Utila, Honduras; RHL: San Salvador, Bahamas; SLC8E: St. Lucie County, FL, USA; VOL: Volusia County, Florida, USA), which were F3 to F6 generations of fish originally collected from the field by Dr. D. Scott Taylor. Fish were isolated within a week of hatching in a laboratory at the National Taiwan Normal University and kept alone in a 13 × 13 × 9 cm^3 ^translucent polypropylene container filled with 550 ml 25 ppt synthetic sea water (Instant Ocean™powder). Fish were kept at 25 ± 2°C on a 14:10-h photoperiod and fed newly hatched brine shrimp (*Artemia*) nauplii daily. Containers were cleaned and water replaced every 2 weeks. Each fish was given a unique identification code. Experiments were conducted in accordance with a protocol approved by The Animal Care and Use Committee of National Taiwan Normal University (permit #96016).

### Experimental design and procedures

This study aimed to investigate whether and if so how competitive ability measured two months previously and contest experience acquired one month previously influenced the importance of the winner and loser effects arising from a one-day-old winning/losing experience. The procedures are outlined in Figure 1. The day the final contests were staged is referred to in this manuscript as 'Test Day'.

This study used naïve fish that were kept isolated from the 7^th ^day after hatching and had never been used in any previous experiments; only fish more than 3 months old and with body length more than 20 mm were used. Two months before Test Day, available fish of the same strain were divided into size-matched pairs (difference in standard length, from the tip of the snout to the caudal peduncle, ≤ 1 mm) and the two individuals of the pairs were allowed to fight until a clear winner and loser emerged (i.e., a self-selection procedure, described below). Bégin et al. [26] demonstrated that a self-selected winner has a 0.83 chance of having a better fighting ability than a self-selected loser. The self-selected winners and losers from these contests were therefore classified as better and worse competitors, respectively. This process is referred to in this manuscript as the '2-month competitive ability' procedure.

One month (30 days) after the 2-month competitive ability procedure (one month prior to Test Day), half of the better competitors were randomly selected to receive a winning experience (1-month winners) and the other half a losing experience (1-month losers) (for details see below). The same procedure was applied to the worse competitors. The experience acquired from this random-selection procedure is referred to as the '1-month winning/losing experience'. Four groups of individuals resulted from the combination of the self-selection and the random-selection procedures thus far.

One month after the 1-month winning/losing experience, the individuals of each of the four groups were again divided into pairs matched for strain and body length. These pairs were randomly assigned to either W-N or L-N contest types. One animal from each pair was randomly assigned to be the focal individual and receive the relevant experience (W or L) one day prior to Test Day and the other to be the control opponent and receive no experience (N) at the same time. This process is referred to in this manuscript as the '1-day winning/losing experience'. The two individuals then fought with each other on Test Day. The differences between the W and the N individuals in initiating aggressive acts and winning contests measure the significance of the 1-day winner effect; those between L and N individuals measure the 1-day loser effect.

Overall, the experiments involved eight different treatments (2 '2-month competitive-ability' types × 2 '1-month experience' types × 2 '1-day contest' types). Five different strains of the fish were used, and 12 contests were staged for each treatment for each strain. The experiment therefore used 480 pairs of fish (i.e., 960 distinct fish). Each fish was used only once in this study.

### Providing a randomly selected winning/losing experience

To ensure that a fish received its pre-designated losing (or winning) experience (in either the 1-month winning/losing experience or the 1-day winning/losing experience), it fought against a much larger/smaller (difference in SL > 2 mm) individual that had won/lost several fights with similar-sized opponents. Experience training was carried out in a 12 × 8 × 20 cm^3 ^standard aquarium divided by an opaque partition into two equal-sized symmetrical compartments. One fish was placed in each compartment, selected at random. After 15-min acclimatization, the partition was removed to allow the fish to interact. Experimental individuals acquired their pre-designated experiences quickly (median < 60 s) and the partition was reinserted immediately afterwards to separate the two fish. Fish assigned to receive no (N) experience were treated exactly as above, at the same time as their assigned contest opponents, except with no trainer in the other compartment. After receiving their pre-assigned experiences, the fish were replaced in their maintenance containers and fed newly hatched brine shrimp.

### Staging contests on Test Day

Four hours after receiving their 1-day winning, losing or control experiences the fish were individually identified by using a needle to break the thin membrane between two soft-rays in either the upper or lower margins (randomly assigned) of the two contestants' caudal fins. The marking procedure did not cause bleeding or observable adverse effects upon the fish's health or behavior and the membranes usually grow back completely in less than three days. After being marked, the two contestants were placed in a standard aquarium containing water 13 cm deep and 2 cm of gravel to acclimatize for approximately 20 h - one randomly assigned to each of two equally-sized compartments separated by an opaque partition. On Test Day, a contest began when the partition was lifted. The two fish of a contest pair were then allowed to interact until a clear winner and loser emerged (see below), subject to a maximum of 1 h. At the end of the contests the partition was re-inserted to separate the two opponents. All contests were videotaped for behavioral analysis.

### Contest behavior

After the partition was removed, the fish usually oriented and moved toward each other, which was considered the beginning of contest interactions. The individual that first erected its gill cover during display was the gill-display initiator. The individual that first launched attacks by rapidly swimming toward and pushing against or biting its opponent was the attack initiator. The time interval between the beginning of contest interaction and the first gill display was defined as the latency to gill display, while the time interval between the beginning of contest interaction and the first attack was the latency to the first attack. A contest was "escalated" if resolved after some periods of mutual attacks or "non-escalated" if resolved with no mutual attacks (i.e., after only displays or a single attack). Escalation duration was the time interval between the first attack and the loser's first retreat. The individual that persistently retreated from its opponent's displays/attacks for 5 min without retaliating was the loser of the contest. A loser's opponent was the winner. If neither opponent initiated attacks and no obvious winner and loser were observed in 1 h, the contest was terminated and classified as "unresolved".

### Statistical analyses

To test whether and if so how the 2-month competitive ability and the 1-month winning/losing experience affected the importance of the winner and loser effect arising from the 1-day winning/losing experience, multiple logistic regression models (likelihood ratio *χ^2 ^*statistics) were used to examine their influences on the probability of the focal individuals (i.e., W individuals in the W-N contests and L individuals in the L-N contests) initiating both gill displays and attacks and winning non-escalated contests. The probability of winning escalated contests was excluded from this part of the analyses because the outcomes of these contests are expected to be mainly determined by contestants' intrinsic competitive ability rather than by previous winning or losing experience [17,21]. Because 2-month competitive ability and 1-month winning/losing experience could affect winner and loser effects in different ways, contest type (based on 1-day winning/losing experience) and the two way interactions between contest type and competitive ability and dominance experience were included in the models. When the two-way interactions were significant, binomial tests were used to examine the significance of winner and loser effects for contest pairs with different competitive ability or contest experiences.

We also examined whether 2-month competitive ability and the 1-month winning/losing experience had any effect on an individual's behavior on Test Day. Multiple linear regressions (*F *statistics) were employed to analyze their influences on escalation duration and the latencies to both gill display and attack (all natural log transformed). Contest type (W-N or L-N), the two-way interactions between contest type and competitive ability and dominance experience were included in the models as they could also influence these measurements.

In all regression models, the mean standard length and strain type of the contest pairs were included as control factors. JMP (v. 5.0.1 SAS Institute Inc,. Cary, NC, USA), a commercial statistical package, was used for the statistical analyses.

## Competing interests

The authors declare that they have no competing interests.

## Authors' contributions

YH conceived of the study, designed the experiments, contributed to data analysis and the manuscript. YTL executed the experiment and contributed to data analysis and the manuscript. Both authors read and approved the final manuscript.
